# Supplementary material for: Systematic Investigation of the Role of Molybdenum and Boron in NiCo-Based Alloys for the Oxygen Evolution Reaction
Source: Molecules. 2025 Apr 29;30(9):1971. doi: 10.3390/molecules30091971 (PMC12074284; doi:10.3390/molecules30091971)
Supplement: Supplementary file 1 [file molecules-30-01971-s001.zip › molecules-3590559-supplementary.pdf]

Supplementary Materials:

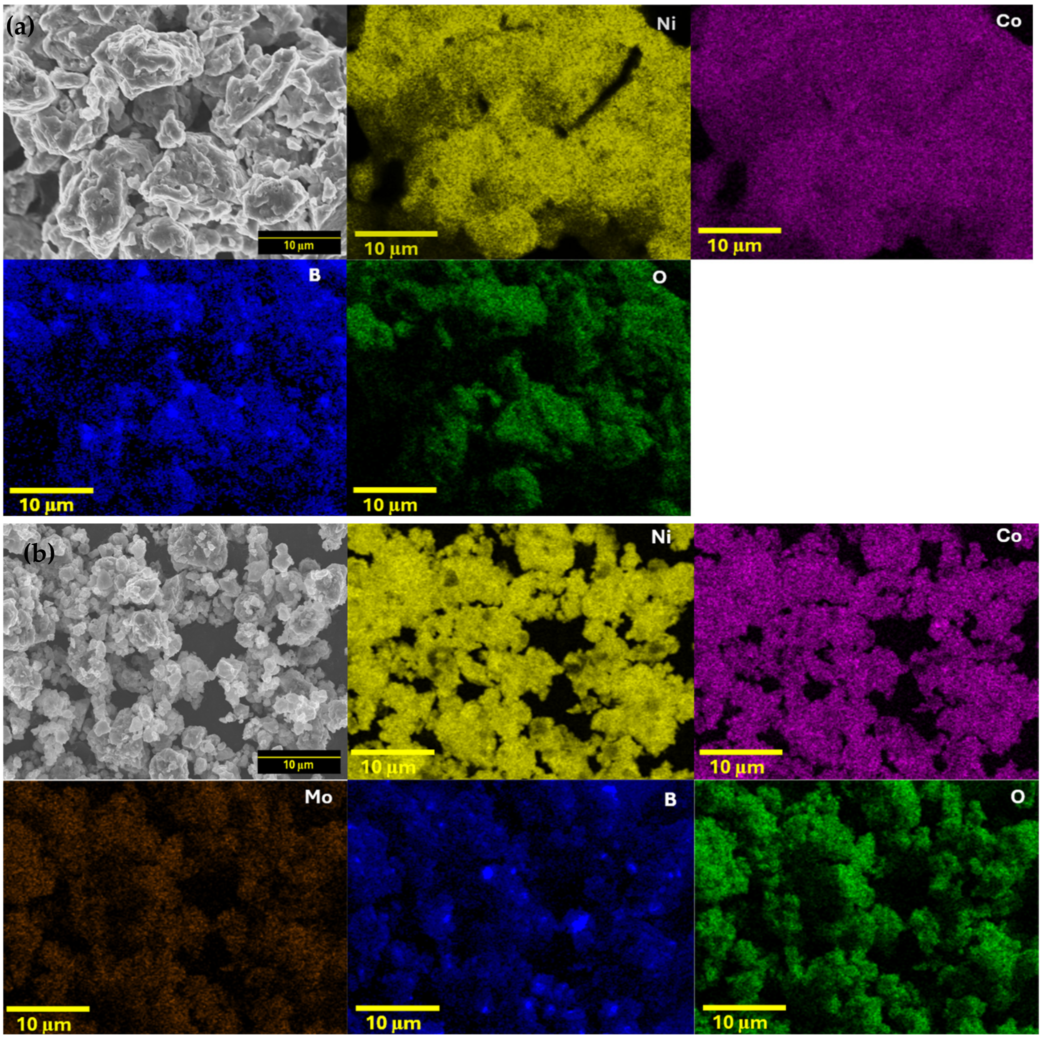

Figure S1. SEM images and EDS mapping of as-cryomilled (micropowder) a) NiCoB and b) $NiCoMo_xB_{1-x}$.

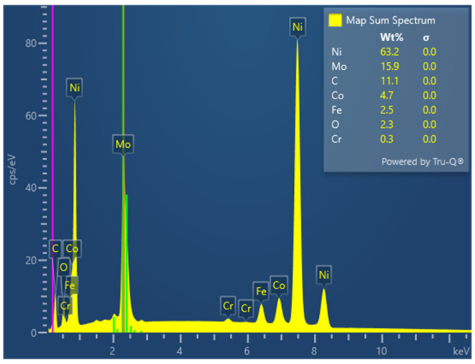

Figure S2. EDS spectra of NiCoMoyBy-SA.

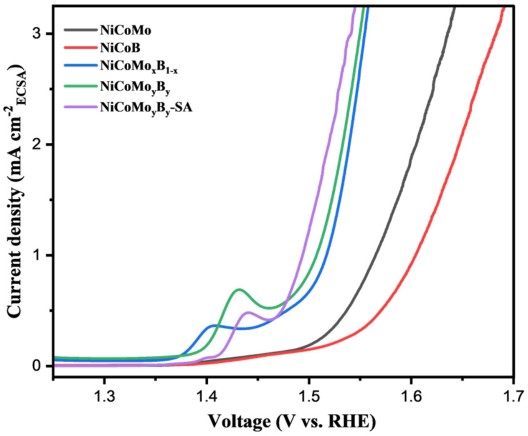

Figure S3. Polarization curve normalized by electro active surface area (ECSA)

Table S1. Ni 2p XPS fitting parameters for $NiCoMo_yB_y$-SA alloy before and after electrochemical testing.

Peak	Pre-OER BE (eV)	Pre-OER FWHM (eV)	Post-OER BE (eV)	Post-OER FWHM (eV)	ΔFWHM (eV)
Ni 2p	852.6 ± 0.0	1.5 ± 0.0	853.4 ± 0.1	1.8 ± 0.1	+0.3
	855.6 ± 0.0	2.8 ± 0.1	855.5 ± 0.1	2.0 ± 0.1	-0.8
	858.2 ± 0.1	2.5 ± 0.2	856.9 ± 0.5	2.4 ± 0.5	-0.1

Peak	Pre-OER BE (eV)	Pre-OER FWHM (eV)	Post-OER BE (eV)	Post-OER FWHM (eV)	ΔFWHM (eV)
	861.0 ± 0.1	3.7 ± 0.1	861.4 ± 0.0	4.7 ± 0.1	+1.0
	869.8 ± 0.0	1.7 ± 0.0	870.8 ± 0.2	1.6 ± 0.3	-0.1
	873.2 ± 0.2	3.1 ± 0.2	873.2 ± 0.1	2.6 ± 0.2	-0.5
	876.5 ± 0.3	4.1 ± 2.4	875.3 ± 0.4	2.1 ± 0.4	-2.0
	879.8 ± 0.2	2.5 ± 0.8	879.4 ± 0.1	6.5 ± 0.1	+4.0

Table S2. Co 2pXPS fitting parameters for $NiCoMo_yB_y$-SA alloy before and after electrochemical testing.

Peak	Pre-OER BE (eV)	Pre-OER FWHM (eV)	Post-OER BE (eV)	Post-OER FWHM (eV)
Co 2p	780.0 ± 0.0	6.0 ± 0.1	778.5 ± 143.2	3.9 ± 124.9
	781.2 ± 0.0	2.0 ± 0.2	780.3 ± 0.2	2.7 ± 0.5
	784.6 ± 0.1	6.8 ± 0.3	782.7 ± 1.3	5.2 ± 5.9
	789.9 ± 0.2	2.4 ± 0.5	787.3 ± 1.8	4.5 ± 1.4
	797.0 ± 0.0	2.4 ± 0.1	796.1 ± 0.0	3.1 ± 0.1

Table S3. Mo 3d XPS fitting parameters for $NiCoMo_yB_y$-SA alloy before and after electrochemical testing.

Peak	Pre-OER BE (eV)	Pre-OER FWHM (eV)	Post-OER BE (eV)	Post-OER FWHM (eV)
Mo 3d	229 ± 0.0	0.67 ± 0.0	-	-
	232.1 ± 0.1	0.67 ± 0.1	-	-
	229.2± 0.0	1.65± 0.0	228 ± 0.0	1.28 ± 0.1
	232.3 ± 0.1	1.65 ± 0.0	231.1 ± 0.0	1.28 ± 0.1
	231.2± 0.1	1.8 ± 0.1	229.8 ± 0.3	1.12 ± 0.0

Peak	Pre-OER BE (eV)	Pre-OER FWHM (eV)	Post-OER BE (eV)	Post-OER FWHM (eV)
	234.3± 0.0	1.8 ± 0.2	233.6 ± 0.1	1.12 ± 0.2
	233.4 ± 0.0	1.29 ± 0.1	232.2 ± 0.1	1.2 ± 0.1
	236.5 ± 0.1	1.29 ± 0.1	235.4 ± 0.2	2.0 ± 4.1

Table S4. B 1s and O 1s XPS fitting parameters for $NiCoMo_yB_y$-SA alloy before and after electrochemical testing.

Peak	Pre-OER BE (eV)	Pre-OER FWHM (eV)	Post-OER BE (eV)	Post-OER FWHM (eV)
B 1s	187.84± 0.1	2± 0.1	192.5±0.1	1.7± 0.0
	189.54± 0.0	1.6± 0.05	189.99±0.2	1.48±0.2
	190.71± 0.0	1.8± 0.0	191.99±0.1	1.21±0.0
	192± 0.1	2.7± 0.1	191.15±0.0	0.97±0.0
O 1s	529.35± 0.0	1.42± 0.0	529.86± 0.0	1.43± 0.1
	530.21 ± 0.3	1.67± 0.2	531.0± 0.0	1.55 ± 0.2
	531.42± 0.1	1.73± 0.4	531.80 ± 0.1	1.49 ± 0.1
